# Myocardial infarction due to coronary embolism after mechanical aortic valve replacement: a case report

**DOI:** 10.1186/s13019-024-02556-7

**Published:** 2024-03-11

**Authors:** Mukan Kagan Kus, Anil Karaagac, Burak Bozkurt, Ufuk Sadik Ceylan, Mehmet Kaplan

**Affiliations:** 1 Department of Cardiovascular Surgery, Dr Siyami Ersek Thoracic and Cardiovascular Surgery Training and Research Hospital, Istanbul, Turkey; 2 Department of Cardiology, Dr Siyami Ersek Thoracic and Cardiovascular Surgery Training and Research Hospital, Istanbul, Turkey

## Abstract

Prosthetic valve thrombosis (PVT) in aortic valve and its complication coronary embolism is a very rare condition. Diagnosis and treatment process is challenging. We present a young patient with acute myocardial infarction who underwent mechanical aortic valve replacement (AVR) operation one month earlier. Percutaneous coronary intervention was performed and it was succesful. Transeasophageal ecocardiography (TEE) was performed. Thrombus was seen on the mechanical aortic valve and thrombolytic therapy was initiated. Control TEE was performed and there was no signs of thrombi. The patient was discharged healthfully with anticoagulant and antiaggregan.

## Introduction

Prosthetic valve thrombosis is rare but life-threatening complication and seen mostly in mitral valve (75%) [1]. Clinical presantations may vary depending on where the embolism occurs. It may be stroke, retinal emboli, mesenteric ischemia, leg ischemia, myocardial enfarctus(MI) and etc. Coronary embolism is a rare complication after aortic valve replacement. There are many causes of this phenomenon. Coronary embolism may result from calcific aortic root plaque embolism, prosthetic valve thrombosis, infective endocarditis, atrial fibrillation(AF) with ineffective international normalization ratio (INR) in valvular-AF patients, AF with inadequate use of anticoagulants in non-valvular AF patients, dilated cardiomyopathy and mural thrombi [2]. There are reported cases in literature that non-atherosclerotic coronary occlusion along with prosthetic valve thrombosis due to subtherapeutic warfarin therapy. We report a 39 years old male patient who underwent a prosthetic AVR one month earlier presented to acute myocardial infarction and was succesfully treated. He has effective INR value (3.0) in admission.

## Case

**Fig. 1:**
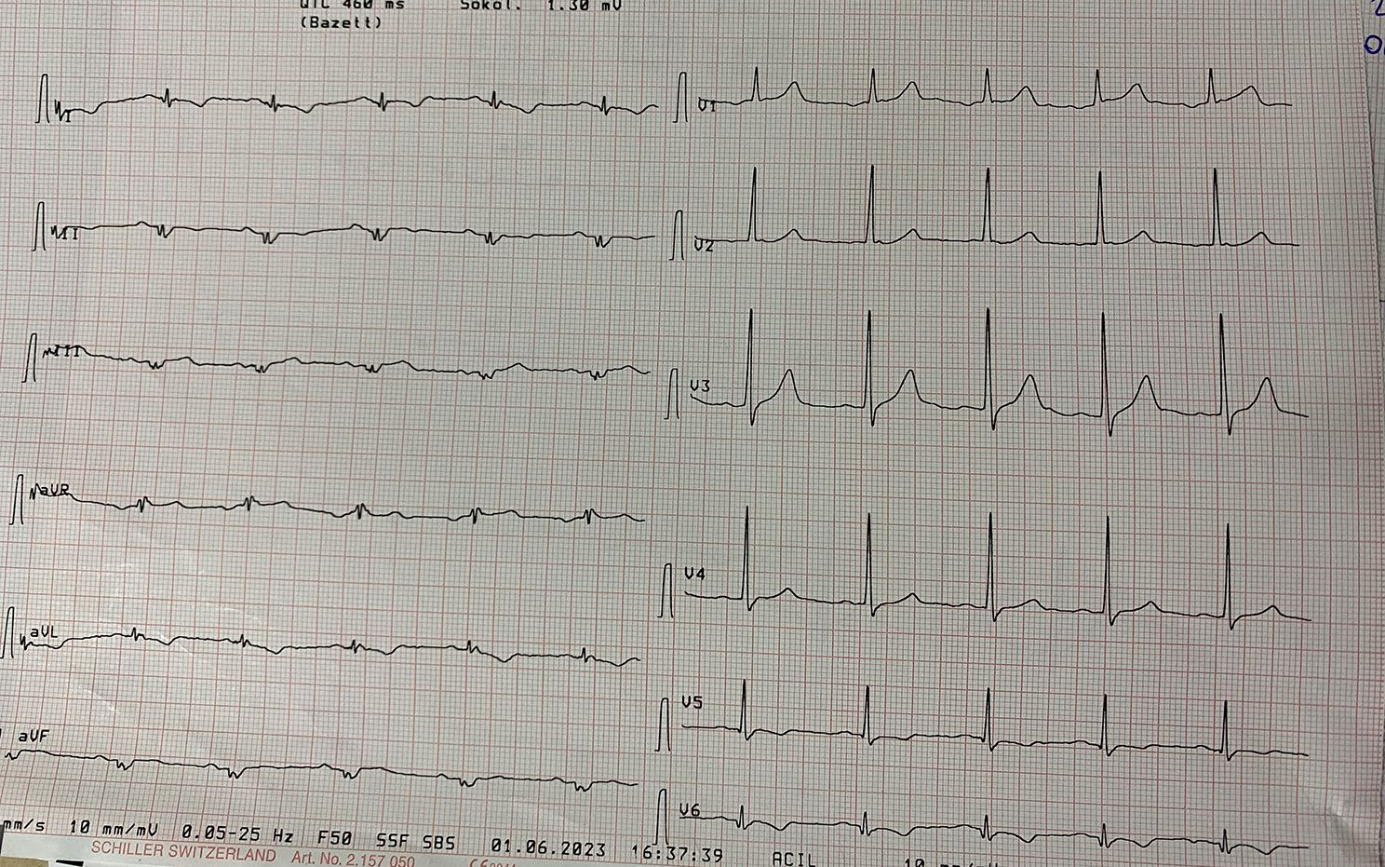
ECG of first admission

**Fig. 2:**
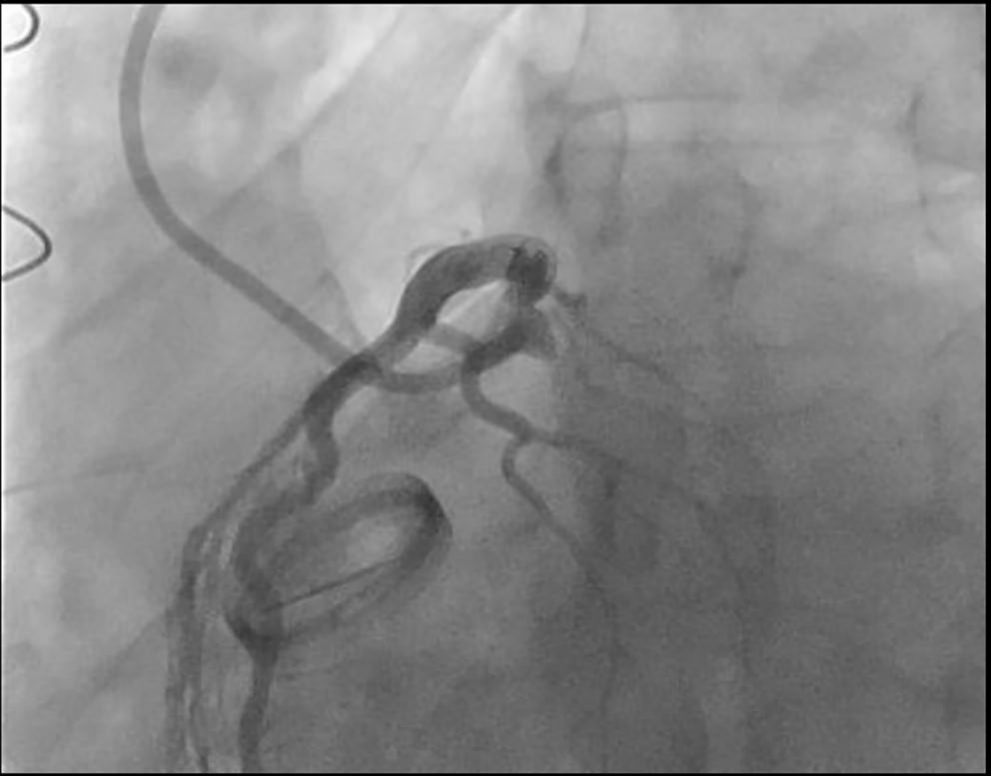
Total occlusion of Circumflex coronary artery (white arrow)

A 39 years old male patient was complaining about dyspnea and palpitation with effort. Transthoracic ecocardiography showed severe aortic insufficiency with rheumatic trileaflet aortic valve. We perform an AVR operation (23 no Carbomedics) through median sternotomy, ascendan aorta, right atrium cannulation and aortic cross clamp. Aortic valve was trileaflet and elongated cusps with no calcification was seen in peroperative assesment. His angiogram was normal before cardiac operation. He was admitted to our emergency room with sudden onset chest pain one month after the procedure. Vital signs demonstrated body temperature 36.5 °C, heart rate 72/min, blood pressure 110/70 mmHg and respirations 20/min. ECG was normal sinus rhtym and has no remarkable ischemic evidence (Fig. 1). Echocardiography was performed, aortic valve was functional, ejection fraction was normal and there was no sign of aortic dissection. We perform a thoracal CT-scan, we suspect aortic dissection because of characteristic and severity of pain and it was normal. High sensitive troponin I level was significantly elevated so the patient was diagnosed with non-ST elevation myocardial infarction (NSTEMI). Coronary angiography was performed and it showed us total circumflex coronary artery (Cx) occlusion (Fig. 2).

**Fig. 3:**
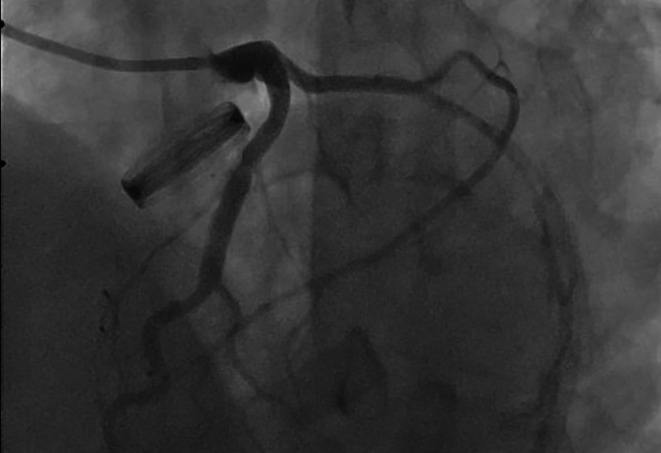
Stent implantation and succesful revascularization of Circumflex coronary artery

**Fig. 4:**
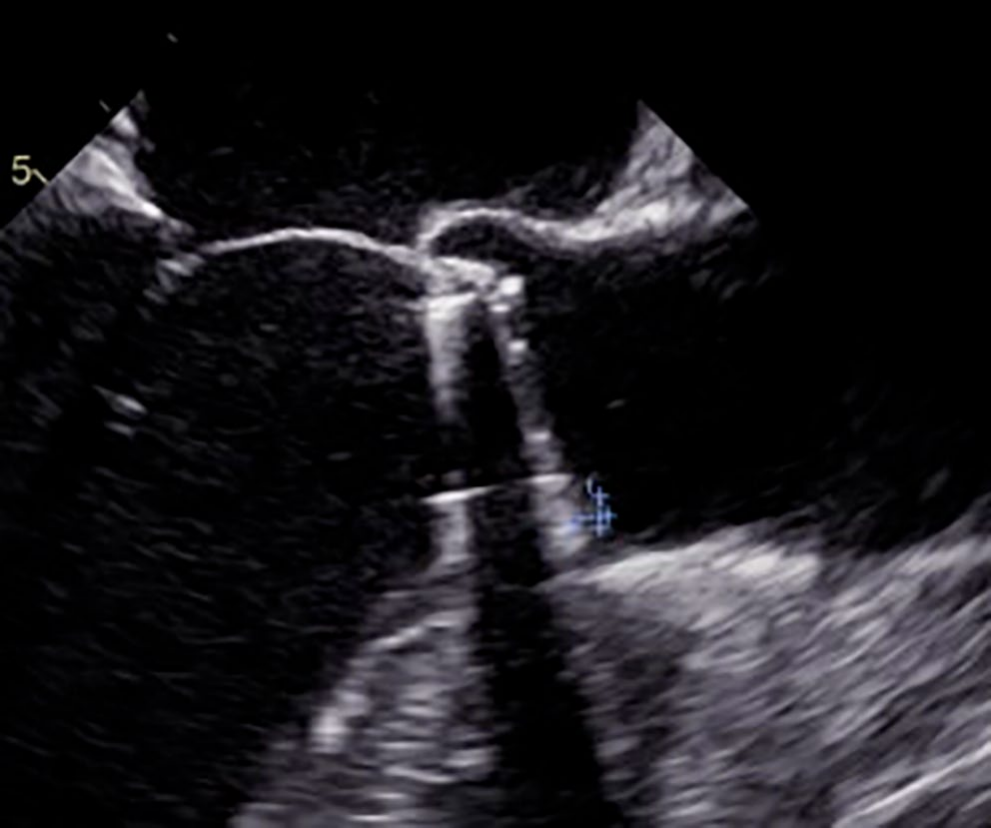
TEE image 3x3 mm thrombus on aortic valve (white arrow)

Coronary balloon angioplasty and stent implantation was performed (Fig. 3). After coronary intervention, the patient was transferred to intensive care unit and his hemodynamics were stable. TEE was planned for detecting thrombi, vegetation or pannus formation on the mechanical aortic valve. TEE demonstrated normal functional bileaflet mechanical aortic valve but there was a 3 × 3 mm hypoecogenic, mobile mass that look alike thrombi formation (Fig. 4). Intravenous thrombolytic therapy (alteplase infusion) was administered in intensive care unit. Control TEE performed 3 days after and it showed there was no thrombus formation like before.

**Fig. 5:**
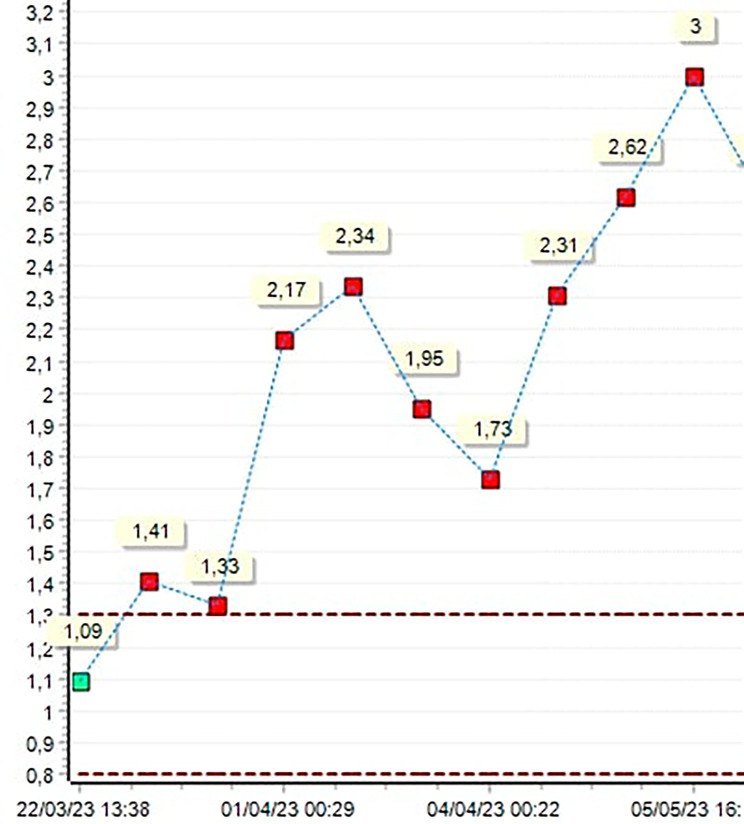
INR values in one month after operation

When we investigate the statistical datas of patient’s INR values along postoperative 1 month, there was two suptherapeutic values of INR (1.95 and 1.73 respectively) consecutive just for 2 days, on postoperative third and fourth days. The patient was receiving warfarin combined with low molecular weight heparin (LMWH) (therapeutic dose) on these days. INR was 2.31 on the fifth postoperative day and LMWH was stopped on that day (Fig. 5). The patient was discharged healthfully with dual antiaggregan (asetilsalisilic asid and clopidogrel) added to warfarin therapy.

## Discussion

Mechanical heart valves are thrombogenic and their use requires that the patient receive anticoagulants [3, 4]. Prosthetic valve thrombosis (PVT) is defined as existence of any thrombus material, in the absence of infection, adhesive or near to the mechanical valve leaflets [1]. Clinical presentation varies from insidious dyspnea to cardiac arrest. It can make a way for catasthropic consequences via occluding blood flow through the left ventricular outflow for aortic valve, diastolic disfunction for mitral valve or embolism to any part of body. Coronary embolism should always be considered in the time of examinating patients with sudden onset chest pain who underwent mechanical valve replacement, especially AVR. It is an uncommon cause of acute myocardial infarction. Coronary embolism is a challenging condition to treat and there is no consensus on management so require individualized therapeutic approach [5]. Embolic material also has tendency of going into left coronary system, because of the preferential flow dynamics due to morphology of sinus valsalva [6].

Dürrleman et al. investigate 5430 patients undergone valve operations in their clinic for 20 years and 39 patients presented with PVT [1]. Previous reports shows that coronary embolism from mechanical aortic valve mostly related to subtherapeutic warfarin therapy [7]. There are reported cases related to patients who abruptly discontinued warfarin therapy, concomitant risk factors like oral contraceptive using or smoking. In our case there was none of these risk factors. Unusual and challenging point about our case is patient has effective INR value in admission.

PVT can be treated with anticoagulants, thrombolytics or surgery. Current guidelines suggest that small (< 10 mm) thrombus on prosthetic valves do not require surgical intervention. We saw that our thrombolytic treatment for 3 mm thrombus is enough. Antiagregant treatment added to warfarin therapy in discharge [8].

In this report we aimed contribution to literature about coronary embolism after mechanical valve replacement, share our first time experience and raise awareness that it can occur in safe therapeutic doses of anticoagulants.

## Data Availability

Datas in the text is acceptable at our hospital and national database system.

## References

[1] Dürrleman N (2004). Prosthetic valve thrombosis: twenty-year experience at the Montreal Heart Institute. J Thorac Cardiovasc Surg.

[2] Kiernan TJ, Ann Marie O, Flynn, Kearney P. Coronary embolism causing myocardial infarction in a patient with mechanical aortic valve prosthesis. Int J Cardiol 112.2 2006;E14–6. 10.1016/j.ijcard.2006.01.038 16814882

[3] Hammermeister KE (1993). A comparison of outcomes in men 11 years after heart-valve replacement with a mechanical valve or bioprosthesis. N Engl J Med.

[4] Cannegieter SC, Rosendaal FR, Briet E (1994). Thromboembolic and bleeding complications in patients with mechanical heart valve prostheses. Circulation.

[5] Shibata T et al. Prevalence, clinical features, and prognosis of acute myocardial infarction attributable to coronary artery embolism. *Circulation* 132.4 2015;241–250. 10.1161/CIRCULATIONAHA.114.015134 26216084

[6] PRIZEL KATEROTHKO, HUTCHINS GROVERM, BULKLEY BERNADINEH (1978). Coronary artery embolism and myocardial infarction: a clinicopathologic study of 55 patients. Ann Intern Med.

[7] Ananda RA, Zhang Z (2023). Coronary embolism due to probable clinical bioprosthetic aortic valve thrombosis: a case report. BMC Cardiovasc Disord.

[8] Mahindru S (2021). Mechanical prosthetic valve thrombosis in current era: 5-year follow-up. Indian J Thorac Cardiovasc Surg.

